# Clinical Usefulness of Standardized Uptake Value Normalized to Lean Body Mass Measured Using a Body Composition Analyzer

**DOI:** 10.7759/cureus.85089

**Published:** 2025-05-30

**Authors:** Tetsu Nakaichi, Kazuaki Funamoto, Shozo Yamashita, Haruki Yamamoto, Kunihiko Yokoyama

**Affiliations:** 1 Division of Radiation Safety and Quality Assurance, National Cancer Center Hospital, Tokyo, JPN; 2 Division of Radiology, Public Central Hospital of Matto Ishikawa, Ishikawa, JPN; 3 Center for PET Imaging, Public Central Hospital of Matto Ishikawa, Ishikawa, JPN

**Keywords:** 18f-fluorodeoxyglucose positron emission tomography (18f-fdg pet), body composition analyzer, lean body mass, standardized uptake value (suv), sul

## Abstract

Standardized uptake value normalized to lean body mass (SUL) is commonly calculated using predictive equations; however, these equations may not accurately reflect lean body mass (LBM) across diverse populations. This study compared SUL values derived from two conventional predictive equations (Pieterman and Janmahasatian) with those obtained using a body composition analyzer (BCA)-based method in a Japanese population and evaluated their clinical utility. Mean SUVs were measured in the liver using a 1-cm diameter region of interest on PET images from 85 subjects (43 males, 42 females) undergoing PET screening. Four subjects (three males and one female) diagnosed with fatty liver were excluded from the analysis. LBM and SUL were estimated using two equation-based methods and a BCA-based method. Pearson's product-moment correlation was used to assess the relationship between body weight and both SUV and SUL. Agreement between equation-based and BCA-based SUL was evaluated using Bland-Altman analysis (with limits defined by the 2.5th and 97.5th percentiles) and intraclass correlation coefficients (ICC, model 2,1). There were significant positive correlations between body weight and both SUV and SUL for all subjects (all P < 0.001). In males, the correlation for SUV exceeded that for SUL, whereas in females, the equation-based SUL values showed weak or negative correlations with body weight. Multiple comparisons revealed that, for the overall cohort, SUL from Janmahasatian’s method did not differ significantly from that obtained using the BCA-based method, whereas in subgroup analyses by gender, BCA-based SULs were significantly different from both prediction formulas. Bland-Altman analysis indicated minimal median differences between methods: the median difference between BCA-based SUL and Pieterman’s SUL was 0.05 g*mL-1 (-0.07 g*mL-1-0.25 g*mL-1), and between BCA-based SUL and Janmahasatian’s SUL was 0.00 g*mL-1 (-0.12 g*mL-1-0.03 g*mL-1), suggesting negligible systematic bias. The ICC between Janmahasatian’s SUL and the BCA-based method was 0.932 overall (0.945 in males and 0.840 in females), while for Pieterman’s method it was 0.845 overall (0.893 in males and 0.658 in females). In this Japanese cohort, SUL calculated using the BCA-based method was overall comparable to that derived from Janmahasatian’s predictive equation, supporting its viability as an alternative for LBM estimation and SUL calculation. However, subgroup analysis revealed that the BCA-based method performed less reliably in females, highlighting the importance of considering population-specific and gender-related differences in clinical PET studies.

## Introduction

18F-fluoro-2-deoxy-D-glucose positron emission tomography (18F-FDG PET) is an imaging method that exploits the enhanced glucose metabolism of malignant tumors relative to normal tissues. It is widely used for incidental lesion detection, staging, tumor quality assessment, prognosis prediction, and evaluation of treatment efficacy [1-3]. In addition to qualitative visual assessment, quantitative evaluation using standardized uptake value (SUV), a semi-quantitative measure, has been performed. Notably, SUV_max_, which reflects the maximum tumor activity, is recognized as a valuable biomarker for many cancers [4, 5]. SUV is calculated as the normalized concentration of FDG based on the administered dose per body weight, assuming uniform tracer distribution. In obese patients, SUV may be overestimated since FDG uptake in adipose tissue is limited during fasting states [6,7].

To address this limitation, SUL-administered dose normalized to lean body mass (LBM) has been introduced as a weight-independent index [7-9]. The PET Response Criteria in Solid Tumors (PERCIST) 1.0 [10] recommends using liver SUL_mean_ as a quality control standard in clinical studies. LBM can be estimated using equations that incorporate parameters such as height, weight, sex, and age; several methods have been proposed [8, 11-15]. FDG PET/CT: European Association of Nuclear Medicine (EANM) procedure guidelines for tumor imaging: version 2.0 introduces the estimation method of Janmahasatian et al. [15] and reports good estimation accuracy for patients weighing greater than 120 kg [16]. Chan compared the technique using the five estimation formulas with the gold standard method using CT and found the method of Pieterman et al. [14] to be most consistent [17]. However, these estimation formulas, derived from diverse populations [18, 19], may not be universally applicable [13, 20]. Moreover, LBM calculated from estimation equations has been shown to differ significantly from LBM measured directly by dual-energy x-ray absorptiometry (DXA), a standard method [21].

Alternatively, a body composition analyzer (BCA), which is integrated with a weight scale and employs bioelectrical impedance analysis (BIA), can measure LBM simply and directly. The high correlation between BCA-derived body fat percentage and that obtained by DXA suggests that the BCA may allow for accurate and straightforward calculation of LBM and SUL [22-24].

The purpose of this study was to compare LBM and SUL calculated using the BCA with those determined by conventional estimation formulas and to evaluate their clinical usefulness.

## Materials and methods

Subjects

A total of 85 subjects (43 males and 42 females) who underwent PET/CT examinations for health screening at our hospital from January 2018 to December 2020 were included in the study. Four subjects (three males and one female) diagnosed with fatty liver were excluded from the analysis. None of the subjects had diabetes or other complications. The mean height was 164.2 ± 8.4 cm (male: 170.5 ± 5.5 cm, female: 157.8 ± 5.6 cm), the mean weight was 63.2 ± 12.7 kg (male: 70.7 ± 12.4 kg, female: 55.7 ± 7.5 kg), and the mean BMI was 23.3 ± 3.6 kg*m^-2^ (male: 24.3 ± 3.9 kg*m^-2^, female: 22.4 ± 3.0 kg*m^-2^). Fasting blood glucose averaged 103.7 ± 11.7 mg*dL^-1^ and remained below 150 mg*dL^-1^ in all subjects. The FDG dose was 4.4 ± 0.3 MBq*kg^-1^, and the body position during PET/CT imaging was from the parietal to the knees with the arms hanging down. Consent for the use of the examination data was obtained from all subjects, and the study was approved by the Ethics Committee of our hospital (No. 30-15).

PET/CT

The PET/CT system was a Discovery ST (GE Healthcare, Milwaukee, WI, USA) with a PET detector consisting of 10,080 BGO crystals of 6.3 × 6.3 × 30 mm^3^. The field of view (FOV) is 15.7 cm, and the slice thickness is 3.27 mm. Forty-seven images can be acquired per bed, and the coincidence time window is 11.7 ns. PET image reconstruction was performed using three-dimensional ordered-subsets expectation maximization (VUE Point Plus) with two iterations and 30 subsets. The reconstructed FOV is 55 cm, and the matrix size is 128 × 128. The post-processing filter was a Gaussian filter with a full width at half maximum (FWHM) of 4.71 mm, and the CT images were used for absorption and scattering correction. The CT image reconstruction method was filtered back projection, with a reconstructed FOV of 50 cm, a matrix size of 512 × 512, a slice thickness of 3.75 mm, and a slice spacing of 3.27 mm. The tube voltage during CT imaging was 120 kVp, and automatic tube current modulation (10-80 mA) was used. PET reconstruction conditions were optimized based on the “Cancer FDG-PET/CT Imaging Guidelines (2nd Edition)” in Japan [25].

Height measuring scale and body composition analyzer

The height-measuring scales were AD-6228AP (A&D Co., Ltd., Tokyo, Japan) and the BCA was DC-270A (TANITA Corp., Tokyo, Japan), both of which were calibrated once every two years. The BCA used in this study estimates body composition using the BCA method based on the basic data obtained from DXA, and by using multi-frequency measurement, detailed measurement of intracellular and extracellular tissue information is possible.

Data analysis

PET images were analyzed using Syngo.via version VB10B (Siemens Healthcare GmbH, Erlangen, Germany). The volume of interest (VOI) was set in the right lobe of the liver parenchyma, and the mean SUV in the liver was measured using CT images as reference. The SUV and SUL were calculated using the following equations.

$SUV (g/mL)=\frac{count (cps)&frasl;volume(mL)}{Dose (Bq)&frasl;weight (g)}&times;\frac{1}{cross calibration factor (cps/Bq)}$

$SUL (g/mL)=SUV&times;\frac{lean body mass (g)}{weight (g)}$

LBM by the estimation formula method of Pieterman et al. [14] (Eq. 1) and Janmahasatian et al. [15] (Eq. 2) was calculated as described below.

$LBM_{p1} (kg)=weight (kg)-\left\{weight (kg)\times \frac{\left[ 1.2\times BMI(kg/m^{2}) +(0.23\times age)-16.2)\right]}{100} \right\} \text{(male)}$

$LBM_{p1} (kg)=weight (kg)-\left\{weight (kg)\times \frac{\left[ 1.2\times BMI(kg/m^{2}) +(0.23\times age)-5.4)\right]}{100} \right\} \text{ (female), (Eq.1)}$

$LBM_{p2} (kg)=9270\times\frac{weight(kg)}{6680+216\times BMI(kg/m^{2})} \text{(male)}$

$LBM_{p2} (kg)=9270\times\frac{weight(kg)}{8780+244\times BMI(kg/m^{2})} \text{ (female), (Eq.2)}$

The LBM obtained by Eq. 1 is denoted as LBM_p1,_ and the LBM obtained by Eq. 2 is denoted as LBM_p2_. BMI is the weight (kg) divided by the square of the height (m). LBM obtained from the BCA method (LBM_BCA_) was directly measured.

Statistical analysis

The relationship between SUV, SUL, and body weight was assessed using Pearson's product-moment correlation coefficient. Because the paired data did not satisfy the normality assumption (as determined by the Shapiro-Wilk test), non-parametric methods were employed. Differences among the three paired conditions (i.e., LBM_BCA_, LBM_p1_, and LBM_p2_; SUL_BCA_, SUL_p1_, and SUL_p2_) were evaluated using the Friedman test. When the Friedman test indicated significant differences, post-hoc pairwise comparisons were performed using the Nemenyi-Wilcoxon all-pairs test to identify the differing groups. In contrast, the subgroup analyses by gender used parametric methods because normality and equivariance (as determined by the Levene test) were confirmed in the subgroup analyses by gender. If significant differences were found in the repeated measures analysis of variance (RM-ANOVA), post hoc comparisons were made by Bonferroni correction. The results of the analyses leading to the determination of nonparametric or parametric methods are presented in Appendix A. Agreement between LBM_BCA_ (or SUL_BCA_) and LBM (or SUL) determined by predictive equation-based estimation was evaluated using the intraclass correlation coefficient (ICC, model 2,1). Systematic differences between SUL_BCA_ and equation-derived SUL were assessed using Bland-Altman analysis, with confidence limits determined by the 2.5th and 97.5th percentiles. Variables were summarized using the median and interquartile range (Q1-Q3). The confidence interval of Bland-Altman analysis was determined by the 2.5% tile and the 97.5% tile. A p-value of less than 0.05 was considered statistically significant. All analyses were performed separately for all participants, as well as for males and females. Statistical analyses were conducted using R (version 4.4.3, (The R Core Team, R Foundation for Statistical Computing, Vienna, Austria)) and the PMCMRplus package (Pohlert, 2024).

## Results

Correlations

**Figure 1:**
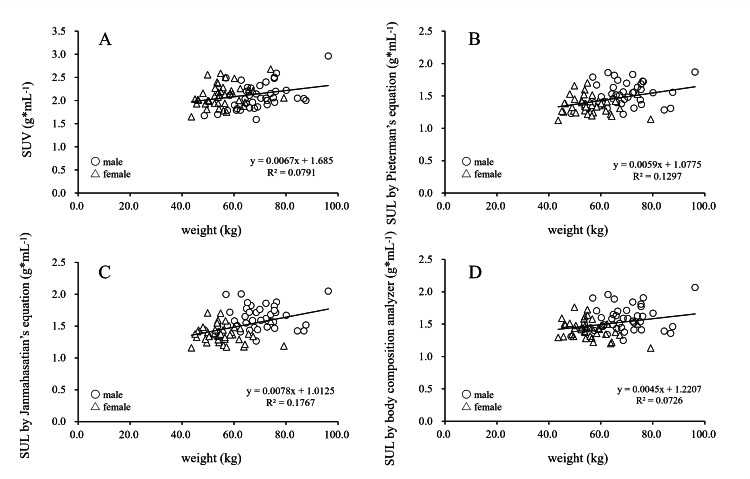
Correlation of SUV and SULs obtained from estimation equations or body composition analyzer to body weight. Scatter plots show a correlation between each SUV (A) and SULs obtained from Pieterman's equation (B), Janmahasatian's equation (C), and the body composition analyzer (D) to body weight. Open circles indicate males, and open triangles indicate females. SUV: standardized uptake value; SUL: standardized uptake value normalized in lean body mass

Figure 1 shows the correlations between body weight and SUV, as well as between body weight and SUL, determined by two estimation equations and the BCA. For all subjects, the correlation coefficients for SUV, SUL_p1_, SUL_p2_, and SUL_BCA_ were 0.28 g*mL^-1^, 0.36 g*mL^-1^, 0.42 g*mL^-1^, and 0.27 g*mL^-1^, respectively (all P < 0.001), indicating positive correlations between body weight and both SUV and SUL. In male subjects, the correlation coefficients for SUV, SUL_p1_, SUL_p2_, and SUL_BCA_ were 0.48 g*mL^-1^, 0.18 g*mL^-1^, 0.13 g*mL^-1^, and 0.18 g*mL^-1^, respectively (P < 0.001, P = 0.263, P = 0.441, and P = 0.263). Although positive correlations were observed, the coefficient for SUV was relatively higher than those for SUL. In female subjects, the correlation coefficients for SUV, SUL_p1_, SUL_p2_, and SUL_BCA_ were 0.30 g*mL^-1^, -0.10 g*mL^-1^, -0.10 g*mL^-1^, and -0.34 g*mL^-1^, respectively (P = 0.055, P = 0.55, P = 0.54, and P = 0.030), with a negative correlation observed only for SUL_BCA_.

LBM and SUL comparisons

Table 1 shows the summary of LBM and SUL. Among all participants, the Friedman test indicated a statistically significant difference among the three LBM conditions (LBM_p1_, LBM_p2_, LBM_BCA_; p < 0.001). The Nemenyi test showed significant differences between LBM_p1_ and LBM_p2_ (p < 0.001) and between LBM_p1_ and LBM_BCA_ (p < 0.001), but not between LBM_p2_ and LBM_BCA_ (p = 0.97). The median (Q1-Q3) values were 41.5 kg (35.0 kg-50.8 kg) for LBM_p1_, 43.6 kg (35.8 kg-53.6 kg) for LBM_p2_, and 41.6 kg (36.9-51.8) for LBM_BCA_. In male subjects, significant differences were observed (p < 0.001) by the RM-ANOVA. All pairwise comparisons were statistically significant with the Bonferroni correction : LBM_p1_ vs. LBM_p2_ (p < 0.001), LBM_p1_ vs. LBM_BCA_ (p < 0.001), and LBM_p2_ vs. LBM_BCA_ (p < 0.001). The median values were 50.8 kg (47.8 kg-53.1 kg), 54.0 kg (51.5 kg-56.7 kg), and 52.8 kg (50.0 kg-55.4 kg), respectively. In female subjects, the RM-ANOVA showed significant differences (p < 0.001). As the results of the Bonferroni post hoc tests, LBM_p1_ differed significantly from LBM_BCA_ (p < 0.001), and LBM_p2_ differed from LBM_BCA_ (p < 0.001), while LBM_p1_ and LBM_p2_ did not differ significantly (p = 0.22). The median values were 35.0 kg (33.1 kg-39.0 kg) for LBM_p1_, 35.8 kg (33.8 kg-37.8 kg) for LBM_p2_, and 36.9 kg (35.4 kg-39.7 kg) for LBM_BCA_.

**Table 1:** The summary of LBM and SUL compariosn among the estimation equations and body composition analyzer. The summary of LBM and SUL comparisons using Pieterman's and Janmahasatian's estimation equations and the body composition analyzer. We expressed the data as the median (2.5th percentile - 97.5th percentile). For all subjects, when the Friedman test indicated significant differences, post-hoc pairwise comparisons were performed using the Nemenyi–Wilcoxon all-pairs test to identify the differing groups. For subgroups by gender, when the RM-ANOVA test indicated significant differences, post-hoc pairwise comparisons were performed using the Bonferroni correction all-pairs test. LBM; lean body mass, SUL; standardized uptake value normalized in lean body mass, BCA; body composition analyzer.

		Pieterman	p-value v.s. Janmahasatian	p-value v.s. BCA	Janmahasatian	p-value v.s. BCA	BCA
LBM (kg)	male (n=40)	50.8 (47.8–53.1)	< 0.001	< 0.001	54.0 (51.5–56.7)	< 0.001	52.8 (50.0–55.4)
	female (n=41)	35.0 (33.1–39.0)	0.22	< 0.001	35.8 (33.8–37.8)	< 0.001	36.9 (35.4–39.7)
	all subjects (n=81)	41.5 (35.0–50.8)	< 0.001	< 0.001	43.6 (35.8–53.6)	0.97	41.6 (36.9–51.8)
SUL (g*mL^-1^)	male (n=40)	1.52 (1.39–1.67)	< 0.001	< 0.001	1.59 (1.49–1.75)	< 0.001	1.56 (1.44–1.70)
	female (n=41)	1.35 (1.27–1.42)	< 0.001	< 0.001	1.37 (1.30–1.44)	< 0.001	1.43 (1.33–1.50)
	all subjects (n=81)	1.41 (1.31–1.55)	< 0.001	< 0.001	1.46 (1.37–1.62)	0.97	1.48 (1.37–1.60)

Among all participants, the Friedman test revealed significant differences among the three SUL conditions (SUL_p1_, SUL_p2_, SUL_BCA_; p < 0.001). Post-hoc comparisons indicated significant differences between SUL_p1_ and SUL_p2_ (p < 0.001) and between SUL_p1_ and SUL_BCA_ (p < 0.001), but not between SUL_p2_ and SUL_BCA_ (p = 0.97). The median (Q1-Q3) values were 1.41 g*mL^-1^ (1.31 g*mL^-1^-1.55 g*mL^-1^) for SUL_p1_, 1.46 g*mL^-1^ (1.37 g*mL^-1^-1.62 g*mL^-1^) for SUL_p2_, and 1.48 g*mL^-1^ (1.37 g*mL^-1^-1.60 g*mL^-1^) for SUL_BCA_. In male subjects, the RM-ANOVA shows a significant difference (p < 0.001). All pairwise comparisons using the Bonferroni post hoc tests were statistically significant: SUL_p1_ vs. SUL_p2_ (p < 0.001), SUL_p1_ vs. SUL_BCA_ (p < 0.001), and SUL_p2_ vs. SUL_BCA_ (p < 0.001). The median values were 1.52 g*mL^-1^ (1.39 g*mL^-1^-1.67 g*mL^-1^) for SUL_p1_, 1.59 g*mL^-1^ (1.49 g*mL^-1^-1.75 g*mL^-1^) for SUL_p2_, and 1.56 g*mL^-1^ (1.44 g*mL^-1^-1.70 g*mL^-1^) for SUL_BCA_. In female subjects, the overall difference was significant (p < 0.001) with the RM-ANOVA. All pairwise comparisons were statistically significant with the Bonferroni correction: SUL_p1_ vs. SUL_p2_ (p < 0.001), SUL_p1_ vs. SUL_BCA_ (p < 0.001), and SUL_p2_ vs. SUL_BCA_ (p < 0.001). The median values were 1.35 g*mL^-1^ (1.27 g*mL^-1^-1.42g*mL^-1^) for SUL_p1_, 1.37 g*mL^-1^ (1.30 g*mL^-1^-1.44 g*mL^-1^) for SUL_p2_, and 1.43 g*mL^-1^ (1.33 g*mL^-1^-1.50 g*mL^-1^) for SUL_BCA_.

Agreement analysis

**Figure 2:**
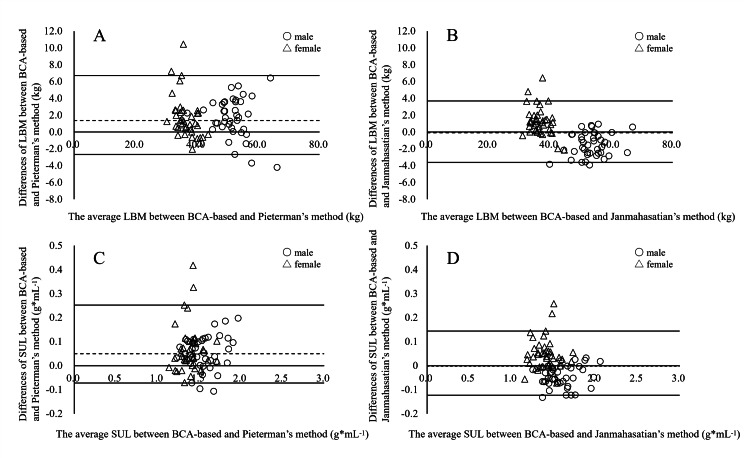
Bland-Altman plot between LBM (SUL) using body composition analyser and LBM (SUL) using estimated equations. Bland-Altman plots were used to compare LBM measured by BCA with LBM calculated using the formula from Pieterman et al. (A) or the formula from Janmahasatian et al. (B). In the same way, SUL measured by BCA was compared with SUL calculated using the formula from Pieterman et al. (C) or the formula from Janmahasatian et al. (D). The upper line in each plot represents the 97.5^th^ percentile value, the middle line the median value, and the lower line the 2.5^th^ percentile value; open circles indicate males, and open triangles indicate females. LBM: lean body mass; BCA: body composition analyzer; SUL: standardized uptake value normalized in lean body mass

Bland-Altman plots (Figure 2) were used to assess systematic differences between LBM_BCA_ (SUL_BCA_) and equation-derived LBM (SUL). For all subjects, the median difference (with 2.5th-97.5th percentile limits) between LBM_BCA_ and LBM_p1_ was 1.37 kg (-2.68 kg-6.70 kg), and between LBM_BCA_ and LBM_p2_ was -0.09 kg (-3.61 kg-3.70 kg). In males, the median differences between LBM_p1_ and LBM_BCA_ and between LBM_p2_ and LBM_BCA_ were 1.84 kg (-3.71 kg-5.48 kg) and -1.68 kg (-3.85 kg-0.77 kg), respectively; in females, these were 1.05 kg (-1.31 kg-7.23 kg) and 1.10 kg (-1.98 kg-4.80 kg), respectively. For SUL, the median difference between SUL_BCA_ and SUL_p1_ was 0.05 g*mL^-1^ (-0.07 g*mL^-1^-0.25 g*mL^-1^) and between SUL_BCA_ and SUL_p2_ was 0.00 g*mL^-1^ (-0.12 g*mL^-1^-0.03 g*mL^-1^) for all subjects. In males, the medians were 0.05 g*mL^-1^ (-0.10 g*mL^-1^-0.19 g*mL^-1^) for SUL_p1_ vs. SUL_BCA_ and -0.05 g*mL^-1^ (-0.12 g*mL^-1^-0.03 g*mL^-1^) for SUL_p2_ vs. SUL_BCA_; in females, they were 0.04 g*mL^-1^ (-0.04 g*mL^-1^-0.33 g*mL^-1^) for SUL_p1_ vs. SUL_BCA_ and 0.04 g*mL^-1^ (-0.06 g*mL^-1^-0.22 g*mL^-1^) for SUL_p2_ vs. SUL_BCA_.

The intraclass correlation coefficient (ICC, model 2,1) between LBM_p1_ and LBM_BCA_, and between LBM_p2_ and LBM_BCA_, was 0.947 and 0.976, respectively (both P < 0.001) for all subjects. For SUL, the ICC between SUL_p1_ and SUL_BCA _and between SUL_p2_ and SUL_BCA_ was 0.845 and 0.932, respectively (both P < 0.001). In males, the ICCs for LBM were 0.862 (LBM_p1_ vs. LBM_BCA_) and 0.920 (LBM_p2_ vs. LBM_BCA_) (P < 0.001 and P = 0.002, respectively), and for SUL, the ICCs were 0.893 and 0.945 (both P < 0.001). In females, the ICCs for LBM were 0.657 (LBM_p1_ vs. LBM_BCA_) and 0.796 (LBM_p2_ vs. LBM_BCA_) (both P < 0.001), and for SUL, the ICCs were 0.658 and 0.840 (both P < 0.001).

## Discussion

In the present study, LBM and SUL obtained from BCA were compared with methods based on Pieterman and Janmahasatian equations for a Japanese cohort. The dependence of variables on body weight was evaluated by correlations, the agreement of SUL between the formula-based method and BCA was examined, and the clinical usefulness of the method was examined. Results showed that, contrary to expectations, the correlation between SUL and body weight was similar to that of SUV for the cohort as a whole, but SUV showed a correlation in men, whereas the gender-specific correlation trend for SUL was weak. Agreement analysis showed that SULs based on BCA were consistent with Janmahasatian SULs, with an overall ICC of 0.932 for SULs (males: 0.945, females: 0.840) and a difference of only 0.1 g*mL^-1^ for SULs in the Bland-Altman plot. While further studies are warranted to address population- and gender-specific factors, the BCA-based method is a direct method for estimating LBM and SUL and shows potential as an alternative to estimating equation-based methods. In addition, the reproducibility of SULs could depend on the reconstruction algorithm (time-of-flight and point spread function) and detector composition (semiconductors), as well as SUVs, and the effects among multiple devices for human subjects should be evaluated.

SUL has been reported as a highly quantitative index that accounts for non-accumulation in theoretical fat and is reported to be less dependent on body weight [7,8,11]. In the present study, the correlation coefficients with body weight for the whole cohort were 0.28 g*mL^-1^ for SUV, 0.36 g*mL^-1^ for SUL_p1_, 0.42 g*mL^-1^ for SUL_p2_, and 0.27 g*mL^-1^ for SUL_BCA_, with no difference between SUV and SUL. In subgroup analysis by gender, SUV showed a correlation coefficient in males, whereas the correlation for SUL was weak. In women, the estimated equation-based method was found to be uncorrelated with body weight, while the BCA-based method was negatively correlated with body weight. In a previous study, the correlation coefficients between body weight and SUV and SUL in the estimated equation of Janmahasatian et al. were 0.58 and 0.04 for men and 0.54 and 0.13 for women, respectively, which were different from our results. The previous study was conducted on Westerners, and differences in race, body type, and number of subjects may have affected the results. In addition, the results may be biased toward certain populations because the analysis was based on screening subjects.

Since direct comparison with the gold standard DXA method was not possible in this study, the validity of the BCA method was evaluated by relative agreement with the methods recommended in the EANM Research Ltd. (EARL) practice guideline [16]. The results showed that the BCA-based LBM and SUL estimates agreed well with those based on the estimation equations of Janmahasatian et al., with ICC (2,1) of 0.976 and 0.932 for LBM and SUL, respectively, with a difference of -0.09 g*mL^-1^ (-3.61 g*mL^-1^-3.70 g*mL^-1^) and 0.00 g*mL^-1^ (-0.12 g*mL^-1^-0.03 g*mL^-1^). However, subgroup analysis by gender showed a statistically significant difference between the two methods, with a slight decrease in the ICC (2,1) of 0.840 (0.796) for SUL (LBM) for females. The discrepancy in LBM and SUL between methods, especially in women, may be related to hormonal balance and menopause, but this study does not have the data necessary to discuss this issue. Previous studies have reported that with the transition to menopause, women simultaneously experience a decrease in basal metabolic rate and lean muscle tissue, which increases the risk of weight gain and obesity [26]. Methods based on simple estimating equations may be inadequate to represent a reversal of the relationship between body weight and lean body mass. Estimated formula methods have been derived from various standard methods and populations [16,17], and it has been pointed out that there may be a large bias for individuals of a different race or body type than the original population [20]. It has also been reported that LBM is significantly lower than body weight in patients with chronic diseases [27], and the BCA method, which can directly measure LBM, may eliminate a larger bias than indirect methods that use height, weight, and sex as parameters to obtain LBM.

Although the BCA used in this study estimated body fat mass using basic data obtained by DXA, it has been reported that DXA overestimates body fat (underestimates LBM) due to fan beam distortion [28]. It is also possible that differences in subjects' body shape in the basic data obtained by the DXA method may have influenced the underestimation of LBM and SUL. Recent reports indicate that BCA, utilizing the four-component method, which assesses fat, mineral, protein, and water content through water mass weighing, heavy water dilution methods, body weight measurements, and the DXA method, can achieve accuracy comparable to the original four-component method [29]. In the future, the estimation of LBM and SUL using the BCA method may establish itself as a gold standard alongside the DXA method.

Because this study focused on healthy screened subjects, the impact on patients with tumors with altered LBM dynamics (i.e., cachexia and sarcopenia) is not clear. Since not only race but also body type, age, and gender (hormonal balance and menopause in women) may have an effect, future studies should be conducted on a larger number of subjects. This study was unable to assess the accuracy of the BCA method due to its lack of comparison with the DXA method. Future research should also evaluate the reproducibility of responses across different imaging systems to guarantee consistent biomarker performance, since SUL can vary with various scanner models and reconstruction algorithms.

## Conclusions

The overall SUL from the BCA-based method in this Japanese group matched closely with the SUL from the Janmahasatian prediction equation in the ICC (2,1) and Bland-Altman tests. This shows that estimating LBM and calculating SUL with BCA is a workable option compared to other methods that rely on estimating equations, as BCA uses bioimpedance analysis, which is a different approach. However, subgroup analyses showed that the BCA-based approach was less reliable for women, highlighting the need to consider differences based on gender and population in clinical PET studies. Additionally, because SUL can fluctuate with different scanner models and reconstruction algorithms, future work must assess response reproducibility across diverse imaging systems to ensure consistent biomarker performance.

## Appendices

Appendix A

Table 2 shows the results of normality tests for LBM and SUL with all subjects and subgroups by gender. All estimation methods confirmed normality in LBM and SUL for each gender, prompting the use of Levene's tests for those groups. The results of Levene's test indicated that the p-value for LBM was 0.90 for males and 0.109 for females, while the p-value for SUL was 0.96 for both males and females. The analysis of LBM and SUL by gender confirmed both normality and the assumption of homogeneity of variance.

**Table 2:** Normality tests for LBM and SUL with all subjects and subgroups by gender Results of the normality test with the Shapiro-Wilk test in all subjects and subgroups by gender for LBM or SUL. An asterisk indicates a statistically significant difference. *: p < 0.05, **: p < 0.01, ***: p < 0.001. LBM: lean body mass; SUL: standardized uptake value normalized in lean body mass; BCA: body composition analyzer

		p-value with Pieterman	p-value with Janmahasatian	p-value with BCA
LBM (kg)	All subjects (n=81)	0.008**	0.001**	<0.001***
	male (n=40)	0.172	0.39	0.162
	female (n=41)	0.78	0.154	0.49
SUL (g*mL-1)	All subjects (n=81)	0.017*	0.024*	0.02*
	male (n=40)	0.36	0.199	0.238
	female (n=41)	0.29	0.128	0.88
